# Strategies used by midwives to enhance knowledge and skill development in midwifery students: an appreciative inquiry study

**DOI:** 10.1186/s12912-024-01784-5

**Published:** 2024-02-23

**Authors:** Fiona Arundell, Athena Sheehan, Kath Peters

**Affiliations:** 1 Western Sydney University, School of Nursing and Midwifery, Building EB LG Room 22, Parramatta South Campus, Locked Bag 1797, NSW 2751 Penrith, Australia; 2 Western Sydney University, School of Nursing and Midwifery, Building 7, G Room 55, Campbelltown Campus, Locked Bag 1797, NSW 2751 Penrith, Australia

**Keywords:** Midwifery student, Clinical practice, Appreciative inquiry, Midwives, Clinical teaching

## Abstract

**Background:**

Midwifery practice experience for midwifery students is an important component of education to enhance knowledge and skill development. Practicing midwives provide student support in the clinical setting, there is minimal literature relating to strategies midwives use to support students.

**Objective:**

To explore midwifery student experiences of the strategies used by midwives to facilitate knowledge and skill development in the clinical practice setting.

**Methods:**

Qualitative approach based on Appreciative Inquiry. The setting is one University in Australia. Participants, thirteen Graduate Diploma in Midwifery students. Individual interviews followed by thematic analysis.

**Results:**

Data analysis identified six themes, Willingness to share knowledge and develop skills; The positive use of questioning; Moderating support; Teaching through the woman; Learning through problematisation and Providing constructive affirmation.

**Conclusions:**

Midwives incorporated varied strategies to support student development in the clinical setting. For an equitable clinical experience, all midwives need support to develop skills and confidence in facilitating student learning.

**Supplementary Information:**

The online version contains supplementary material available at 10.1186/s12912-024-01784-5.

## Background

Midwifery education programs vary internationally; however, they commonly comprise theoretical and clinical practice components. The equal emphasis on theory and midwifery practice experience demonstrates the significance of each component. Midwifery education in Australia is provided as a collaboration between universities and hospitals, and midwifery programs are accredited by the Australian Nursing and Midwifery Accreditation Council (ANMAC). There are two models of midwifery education, postgraduate programs for registered nurses and the Bachelor of Midwifery (BM). This study involved participants undertaking the Graduate Diploma in Midwifery (GDM) a postgraduate midwifery program. This model is for the most part an employed student model where universities provide the theoretical component of the course and hospitals provide employment, clinical education, support and supervision. The midwifery student must meet the standards and clinical skills prescribed by the Australian Nursing and Midwifery Accreditation Council (ANMAC) [1]. Midwifery practice experience provides students the opportunity to apply theory to practice and knowledge, intuition, and reflexivity as it relates to midwifery [2]. Most of the responsibility for supporting the development of these standards and clinical skills is undertaken by practicing midwives, who are allocated to midwifery students on a shift by shift basis.

The acquisition of clinical skills is essential to midwifery student education however, students repeatedly provide disenchanted and negative accounts of their clinical learning experience [3–6]. This suggests that a midwife with professional experience might not necessarily have the skills to effectively facilitate student learning.

When students are supported in the clinical learning environment, confidence and competence are developed [7, 8]. Conversely, midwifery students identify that a lack of commitment and capacity from some midwives to support them in the clinical setting is a source of student stress [9, 10]. Skill development is often reliant upon students observing midwives carrying out procedures, usually with minimal explanation [11, 12]. Development of clinical skills can be so fragmented that students are not able to envisage a holistic approach to care [13, 14]. The lack of support for students in the clinical setting may be linked to midwives being underprepared for role expectations, in Australia the Midwife Standards for Practice state in standard 3.4 that the midwife ‘contributes to a culture that supports learning, teaching, knowledge transfer and critical reflection’ [15]. Midwives have identified an insecurity in supporting the educational development of students [16] and have reported teaching using methods by which they were taught [11]. Midwives however have acknowledged, that if they were adequately educated for the role, student learning would improve [13, 16, 17].

The qualities of an effective clinical teacher include, confidence in their own skills; interest in teaching; providing demonstration-observation-feedback; teaching to suit students’ needs; good communication skills; and awareness of potential learning opportunities [11]. Students respond most positively to midwives who encourage learning by implementing strategies such as questioning [18] and in-practice reflection [18–20].

Although several qualitative studies (3–7; 9–14) have explored the midwifery practice experience of midwifery students, findings primarily highlight the negative aspects of the experience and in particular the relational, social, and emotional experiences of students. While some positive teaching and learning strategies have been identified there is minimal literature detailing the realisation of effective strategies used by midwives to support knowledge and skill development. Therefore, this study sought to explore the midwifery role in the provision of peak experiences for students on midwifery practice experience that had not been explored in previous related studies. The aim of this paper is to report findings that highlight and describe strategies implemented by midwives that students valued as facilitating their knowledge and skill development in the clinical practice setting.

## Methods

### Study design

Previous research has primarily identified the deficits of the midwifery practice experience and in doing so has potentially failed to appreciate supportive practices and behaviours already in place [3–6]. When considering the prominence of negative experiences revealed in previous studies, an alternative methodology was sought to optimize the potential of capturing positive experiences for midwifery students on midwifery practice experience. To highlight optimal examples of student skill and knowledge development in the clinical setting, Appreciative Inquiry (AI) was chosen as the methodology because of its focus on the exploration of positive experiences [21]. AI focuses on what is effective and acknowledges that a solution to improvement already exists [22]. Consequently, this study aimed to discover the current positive strategies and behaviours of midwives to support clinical skill and knowledge development of midwifery students. AI has four phases, identified as the 4D cycle, the four phases include discovery, dream, design, and destiny [21]. The process of discovery provides an understanding of the ‘best of what is’ the dream phase to imagine the ‘what might be’, the design phase constructs the ‘what should be’, the design phase to sustain ‘What will be’ [21].

The focus of this paper was the discovery phase of the study, to provide an understanding of what is being done well [22]. The discovery phase interviews generated individual student’s peak experiences of the strategies implemented by midwives to facilitate knowledge and skill development in the clinical practice setting. The discovery phase provides new detail and insight into peak midwife strategies and behaviours to support midwifery student knowledge and skill development.

### Participant selection and setting

All participants were registered nurses who were enrolled in the GDM and currently on midwifery practice experience at a large tertiary institution in Australia. Participants were personally invited to participate by a midwifery academic not involved in the study or student teaching. Participants were given the option to be interviewed on campus or at a public location of their choosing. All students except one chose to be interviewed on campus on a routine study day, only one student selected to be interviewed at their employing hospital.

### Data collection

Each participant was interviewed two to six months after commencement of clinical placement, using an AI interview guide. The interview guide was developed for this study (see supplementary file). In keeping with AI, the questions were designed to have the following qualities, asked in the affirmative; generated from a primary question that draw upon specific past experiences developed from the topic being explored; encouraged storytelling; appreciated ‘what is’; encouraged the uncovering of valuable experiences [21, 23]. This method of questioning has been compared to the ‘glass half full’ or positive approach compared to the ‘glass half empty’ or negative approach to questioning [22]. The duration of interviews ranged between 40 and 80 min. Questions followed a specific format of lead in questions, followed by topic questions, backward questions which focus on past experiences and concluding with inward questions which discovered the attributes and impact of positive support. The format and rationale for the AI interview and the type of questions asked is described in detail in a previous methodological publication [Authors’ own]. All interviews were audio recorded and transcribed. Participants were offered the opportunity to review their transcript and comment or correct if they wished.

### Data analysis

Data were analysed using the six stages of Braun and Clarke [24] thematic analysis framework. First, the transcript of each participant was read and re-read to gain a deep understanding of the data. Each transcript was annotated to identify patterns, repetitions, differences, and similarities. In the next stage, initial coding (level 1 coding) of the whole data set, the codes were then arranged into themes. The entire data set was then reviewed (level 2 coding) identifying further themes and sub-themes. Themes were discussed and refined with all authors and findings were generated and supported with quotes.

### Ethical considerations

The study was approved by the Institutional Human Research Ethics Committee (H11484). Students were informed about the purpose of the study and requirements of participation. They were assured that their participation would be strictly confidential and voluntary. Participants chose pseudonyms to ensure data were deidentified for use in dissemination and were aware that they could withdraw from the study without consequence.

### Research team and reflexivity

The first author, a midwifery academic, conducted all interviews, but did not have a direct relationship with the study participants at the time. After each interview a reflective journal was completed by the interviewer to reflect on the process and consider whether the interviewer’s preconceptions or the style of questioning during the interview influenced participants’ responses. These reflections were discussed with other authors to ensure rigour in the research process was maintained.

## Results

Thirteen students agreed to participate in the study. All were female, aged between 22 and 50 years, with between one and 25 years’ experience as a registered nurse. All participants were enrolled in a 14-month GDM program involving one day per week of on-campus learning and four days per week employment as a midwifery student in a maternity unit.

Analysis of data identified six themes, *Willingness to share knowledge and develop skills; The positive use of questioning, Moderating support; Teaching through the woman; Learning through problematisation and Providing constructive affirmation.*

### Willingness to share knowledge and develop skills

Students recognised that supportive midwives were knowledgeable and skilled practitioners who shared their knowledge. Midwives who were lifelong learners and used evidence to support practice were admired, instilled confidence in the student, and were considered reassuring, Lilac (p.10) described a midwife’s knowledge as ‘comforting’. As well as sharing knowledge, supportive midwives challenged students about their practice and encouraged them to read evidence and decide how research would influence their future practice. Poppy recalled a conversation with midwives discussing perineal support in labour and their subsequent reaction to her thoughts on the topic.

*There is actually some research about this,…[they say] you should have a look…every time I ask them something they’re very keen to share…They say, oh, I’ve done this research, or I’ve read an article regarding this. That’s why I feel like I should do it this way.* (Poppy, p.5)

Poppy appreciated the opportunity to practice alongside midwives who were enthusiastic about evidence-based practice. The use of evidence in teaching promoted trust in the midwives’ knowledge. Therefore, this knowledge was subsequently transferred to the student and influenced how she envisaged her future practice.

As well as using research evidence, midwives who were willing to expertly explain and demonstrate skills were considered by participants to be more supportive than those who simply role modelled. Students recalled supportive learning experiences that related to both fundamental and complex midwifery skills. Supportive midwives had an appreciation that a fundamental skill might need to be taught more than once therefore building on existing skills. Peony had previously been shown how to undertake a palpation on a woman at term but appreciated being supported to develop the specific skills required for a palpation on a woman at only twenty weeks gestation.

*I had a really lovely midwife…go over a proper palp [palpation] with me. It was the first time I’d actually done a proper palp [palpation] on a 20-week antenatal.* (Peony, p.5)

Students appreciated being sought out to develop less common midwifery skills. Daisy recalls her experience of a midwife including her in the delivery of care for a stillborn baby and their parents.

*Just the practical skills of getting a footprint and a handprint. She was sharing that and things like oh this is good that you’re seeing this as a student.* (Daisy, p.17)

Supportive midwives had the ability to share knowledge through advice and tips grounded in years of experience, for example, prevention of perineal trauma.

*If you’ve got blanching, you know be careful because it might rip. You might have a tear. This is how you would guard the perineum to prevent that. Warm compresses.* (Jasmine, p.7)

Sharing knowledge and skills, also required midwives to consider when and where to do so. Fleur (p.8) recognised that a midwife considered students’ needs by ‘calmly explaining things outside the room’ ensuring the priority in the room was the woman. Similarly, Iris appreciated that the midwife briefed specific aspects of expected care before entering the room.

*We did talk about it before we went in there…We’ll do the Syntocinon in the arm, not the leg, because it’s underwater* (Iris, p.10).

The outcome of the briefing was that the midwife displayed trust in the student to provide autonomous care. *She just left me to it* (Iris, p.10).

### The positive use of questioning

The use of questioning was seen as helpful by students and fell into two categories, ‘student-led questioning’, and ‘midwife-led questioning’. Midwives who took the time to encourage students to ask questions, or asked students questions, were considered to be invested in student learning.

### Student-led questioning

Students who asked questions frequently considered this as taking responsibility for their own learning. Receptive midwives were key to effective student learning because they encouraged and supported the students to ask questions.

*She definitely listens…I’m not embarrassed now to ask her questions because I’m thinking I’m an RN, I need to know these things. She tells me “No you’re still learning and you need to ask”.…she makes me feel comfortable* (Lilac, p.10).

Supportive midwives let students know they were available to answer questions, with statements such as ‘look if you’re not sure come and ask’ (Peony, p.8) and ‘(the midwife) asked me lots of times, do I have any questions’ (Jasmine, p.6). This provided students with confidence to ask questions.

### Midwife-led questioning

Questioning from midwives took several forms including assessing student knowledge on specific topics to establish potential knowledge deficits.

*She went through and asked us what’s that drug for? What do we use it for? Any idea what the standard dose is?* (Rose, p.9)

Students were receptive to this style of learning. They found it to be to the point, reinforcing that this was expected knowledge in the clinical setting. Midwives would also use questioning to ensure students understood what they were going to do in specific scenarios.

*She’d say to me okay this lady now is 28 weeks, what would you be looking at at 28 weeks?* (Lilac, p.6)

When questions were asked in a positive way, even in front of women, students felt comfortable because they were not being asked in a way that made the student look inept. Consequently, there was not a negative impact on the student/woman relationship.

*They were never questions to degrade me. I always felt that she was asking so the woman can feel confident that I knew what I was doing.* (Lilac, p.6)

As students progressed through the course, questioning became more complex, less reliant on recall and more problem based.

*…she’d find time to come back to me and be like, so what have you done? Why did you do that? What do you want to do next?* (Aster, p.9)

Students reported midwives could pose questions creatively. In one example, the student recalled being overwhelmed by completing a CTG interpretation, she did not think she had the skills to undertake the task. The lack of confidence manifested as student negativity and a resistance to knowledge development. The midwife sensed the student response to the situation and then reframed her questioning.

*It was my antenatal rotation and I just said in frustration, and she was right there, and I said I hate CTGs, don’t make me do CTGs I hate them. I mean I walked away I must’ve had a really bad day… She comes around, she plonks herself down and she goes okay, here’s a CTG, tell me what you hate about it. I went okay this is what I hate about it* (Lilac, p.11).

The midwife did not accept avoidance but was creative utilising humour to deflect the student’s negative sentiment in order to develop learning.

### Moderating support

Students described varying levels of support from midwives in the clinical setting. Supportive midwives were able to moderate the level of support provided to the student depending on need, and usually this moved along a continuum from high to low support. When midwives moderated support, it demonstrated to students that there was not a generic approach to support provided but was considered according to a student‘s need and ability. Knowing that a midwife was in close proximity also encouraged students to undertake skills they would not have attempted when in an unsupported environment. High support required the midwife to be close to the student most of the time, often working in such close proximity that many of the tasks undertaken were shared. As student knowledge and skills developed, and support moved to low support the midwife maintained such proximity to the student to be aware of student care provision and available if required, however at a distance that enabled the student to practice independently.

In the early weeks of placement, students reported high support from midwives to meet their learning needs, which students viewed positively.

*I noticed at the beginning when I was with her in clinic days she was sitting right beside me like overviewing everything that I was doing. She was very helpful giving me information, according to the weeks [of gestation] of the woman.* (Lilac, p.5).

Aster also described an intense two-week orientation to antenatal ward, expressing that as a result she had increased confidence in this setting. After initial close supervision, Aster perceived that with her growing confidence, the midwife also had increased confidence in her ability. This allowed Aster to take more responsibility with the midwife close at hand if needed.

*I found it so helpful… She would go through the ward routine and as we had been working together for a few days she’d allow me to take control of the day and manage my time and everything and just step in say, why are you doing this, like rationale everything that we are doing.* (Aster, p.8)

Students recognised that high support and use of positive and encouraging communication pushed them to attempt skills they may have otherwise avoided.

*If there’s any questions don’t worry about it, I’m right here, I’ll help you through it. But I am sure you can do it and we’ll do it together. That support from them to say you can do it, I’ll be here. Doesn’t matter if you mess it up.* (Fleur, p.9)

As knowledge and skills developed there was a transition from high to low support. This transition was most evident in the antenatal clinic, where the midwife/student dyad had the greatest engagement. Unlike in other clinical areas, the long-term student/midwife exposure in the antenatal clinic enabled midwives to have knowledge of the student’s ability.

When experiencing moderated support, students had the confidence to voice the type of support they needed. The boost of confidence provided by moderated support encouraged students to be more proactive in their development and to initiate care independently knowing support was available if required.

*When I work with someone and they will say what are you up to, I’ll say I need this and this and this, I have done this before, but can you just be close by.* (Daisy, p.26)

Being able to independently assess, plan and implement care with the back-up of a midwife was described as ‘exciting’ by Daisy.

*I think the first high point that comes straight to mind is the birth with one of the recent graduates. It was the first normal that I’ve seen in terms of she wasn’t induced. She didn’t have an epidural. Certainly, I was able to do all the things that I’m meant to do, the palpation, the VE (vaginal examination). I did it first and then she did it and then I had to explain to her and we agreed four centimeters, so that was very exciting.* (Daisy (p.7).

For moderating support to be effective a midwife needs to be conscious at all times of a student’s progress through the course and level of capacity.

### Teaching through the woman

Students were aware that midwives’ priority was to meet the care needs of women and babies whilst supporting their development. Students frequently recalled midwives converging the women’s need for midwifery care with student learning, by teaching through the woman. This technique did not compromise the woman’s care whilst providing education for the student. The use of teaching through the woman was either protracted as in a clinic setting where the whole visit incorporated teaching through the woman or included in a single skill or action.

*Instead of making it a separate little learning thing, they just do it as part of the care that’s being given for the woman.* (Rose, p.29)

The practice of teaching through the woman was also seen as being beneficial for women, the focus remained on them while opportunistically providing student education.

*They’re teaching me at the same time as well, so they are including me in the conversation with the woman and the woman doesn’t mind that they’re teaching me at the same time.* (Bluebell, p.19)

### Learning through problematisation

Problematisation occurred when midwives assisted students to recognize problems with clinical practice. There were two types of problematisation. Firstly, a student may have identified a problem themselves but required assistance from the midwife to resolve it. Alternatively, a student may have been unaware that they were facing a clinical problem and needed a midwife to help them identify and resolve it. Students were realistic about their limited knowledge and appreciated midwives’ identification of incorrect knowledge or actions. Regardless of the source of the difficulty students appreciated the positive approach to problem resolution, the midwife would be respectful and kind in their approach. A variety of examples of problematisation were identified.

*I happened to notice one deceleration which probably wasn’t a big deal, but I went out and spoke to the midwife who was actually really positive that I’d come out and I’d spoken to her. She went over why there would have been a deceleration at that point in time. Reassured me that I’d done the right thing by alerting her to the situation* (Peony, p.6).

Students described instances of misinterpreted information where, if it had not been recognised as a problem by a midwife, they would have provided incorrect care. Commonly, supportive midwives identified problems, addressed them in a way that did not highlight student inadequacy to the woman, and were able to address them in appropriate timeframe. For example,

*If… it was the wrong thing I was saying, they would just pipe up and be like or you could try this…Or if I was doing the wrong thing then they would start doing it over the top of me, but then when we left the room, they’d be like you did this wrong… They do rectify it at the time but not in a way that’s…obvious to the woman* (Iris, p.14).

Supportive midwives exposed students to clinical experiences that could challenge their decision making and expose the student to new problems for which they needed solutions. Confidence to develop new skills occurred when midwives provided opportunities for exposure to new experiences while providing gentle guidance and correction if necessary. Aster gave an example of this when undertaking a vaginal examination,

*If I get it wrong, she is really nice about it. She doesn’t go, no, that’s not right. She will be like, no, but have a feel here. Can you feel more cervix here? She will explain a little bit more what I am feeling.* (Aster, p.7)

Although Aster did not expect to get all elements of the vaginal examination correct, she knew the midwife would guide her in developing the required knowledge and skills.

Though a potentially challenging strategy, the consistent message was that the key element of problematisation, was that the midwife involved the student in the process of identifying and resolving the problem.

### Providing constructive affirmation

Students reported the importance of receiving positive feedback on clinical performance, which they appreciated, as this was not a common occurrence. Daffodil described positive feedback from a midwife who reassured her that she was where she should be in terms of skills.

*You’re doing really well. I didn’t have to do much in there. You’re probably where you should be or a bit further on for where you are in your course. She just gave me good feedback and it just made me feel happy about what I’d done and where I’m going.* (Daffodil, p.10)

Students were realistic and accepted that not all feedback on performance would be positive, however as long as it was constructive, receiving feedback was always appreciated.

*She’s very good at constructive criticism, she’ll put it in a way where she’s like I’ll help you work on this and we’ll do that. So next time I see you we’ll do this okay… so a really great person to learn off definitely.* (Bluebell, p.10)

When there was an educational and developmental approach, students welcomed constructive affirmation.

## Discussion

This study used an AI methodology to discover positive strategies used by midwives to support the development of knowledge and skills of midwifery students on midwifery practice experience. Findings from this study identified that midwives who were invested in student learning used a variety of strategies to support the development of knowledge in midwifery students. Although previous research has found that an effective teacher provides learning opportunities such as demonstration-observation-feedback [11] questioning and reflection [18–20], this study provides an understanding of how these strategies are effectively implemented by midwives and the impact on students. This study also identified strategies not previously identified such as Learning through problematisation and Teaching through the woman and Providing constructive affirmation.

Midwives’ willingness to share knowledge and skills, and the use of evidence to support practice, engendered student trust and confidence in the transference of knowledge and that what they were being told was correct. This is important as knowledge development during midwifery practice experience contributes to program completion and confidence and competence as a new graduate midwife [13, 14]. The development from a novice student to a beginning practitioner is identified by Benner [25] as requiring the support of knowledgeable clinicians. Midwives need to be confident in their own practice to be able to support student development, Bäck, Sharma [14] identify the importance for a midwife to be able to practice with confidence to preserve the ability to provide safe woman-centred care.

Midwives in Australia are expected to contribute to the teaching and knowledge development of midwifery students [15] yet literature has indicated that midwives do not know how to fulfill this expectation [13, 15]. In order to effectively support students, midwives recognised the need to keep up to date with practice knowledge to match students’ theoretical knowledge and did this by reading contemporary literature and undertaking further study [17]. Although midwives were allocated to support students on a shift by shift basis, students sought additional support from midwives who had a desire and capacity to share their knowledge based on evidence, which is similar to findings from other studies [6, 7, 18, 26]. The clinical support role of the midwife in relation to supporting the development of midwifery students should be more clearly defined and the knowledge and skills to successfully fulfill this responsibility provided to midwives [8, 27].

Students from the current study described a variety of strategies employed by midwives to support skill development. This study builds on previous research by Chamberlain; Hughes and Fraser; Currie [11, 17, 28] on the merit of strategies such as role modelling and questioning, though these studies were unsupported with a detailed understanding of how these strategies are used by midwives. In this study student data has enhanced the understanding of these strategies by detailing the processes employed by midwives and the student impact when sharing knowledge and skills and the positive use of questioning. The benefit of positive role modelling is that this is likely to be modelled by students [28]. Felstead [29] contradicts Bandura suggesting that because students need to fit in, they may copy behaviour that contradicts the philosophy of midwifery care. Students in this current study chose to align themselves with midwives whose practice they wanted to emulate. Although questioning is a commonly used strategy there is an art to the construction of questions and when to apply various types of questions in the clinical learning environment, this topic has had minimal discussion in relation to midwifery education. This study expanded the understanding of the types of questioning used by midwives and student response to being encouraged to question and being questioned. Although developed as a model for facilitating nursing student learning on midwifery practice experience a narrative approach to questioning has the potential to be applied to midwifery students to develop more personalised responses and subsequent knowledge development [30].

The strategy identified as Moderating Support in this study is comparable to a strategy identified by Zwedberg, Barimani [17] called Fading, a purposeful decrease in the level of support provide to a student, with the goal of independence in practice. Midwives in this study incorporated fading as a strategy in their practice where they withdrew support over time to encourage student independence and initiative. Initially by gaining an understanding of students’ abilities and providing timely and salient learning opportunities, midwives transitioned from providing high to low support. As student competence developed the exposure to complex experiences increased, termed by Spouse [31] as Scaffolding.

It has been argued that traditional strategies of teaching, may not suit the complex scenarios and patterns presented to students in the clinical learning environment [31]. Midwives can act as a conduit to enable an understanding of the complexity of midwifery craft knowledge [31–33]. Participants in this study identified problematisation as an effective teaching strategy used by midwives, in more complex situations. The benefits of problematisation were identified by Titchen [33] however for successful implementation this complex strategy requires confidence in application [32, 33]. This study demonstrated the student benefits of increased confidence to practice knowing that a midwife would solve or identify issues if they occurred.

Teaching through the woman as a creative learning strategy has not been described previously and is a unique finding of this study. In this study, students recognised that for time-poor midwives, this strategy fulfilled the competing requirements of caring for the woman but also supported student learning. What was an effective strategy that enabled midwives to support student learning without compromising care. It could also be hypothesized that the woman also benefitted by becoming more aware of her own care. Incorporation of this strategy by midwives could reduce the burden of the conflicting responsibilities of care provider and educator, it would also reduce the incidence of lost learning opportunities [12].

In this study students reported appreciating feedback from midwives that provided opportunity to reflect on their development. As found in previous studies students were aware that feedback and subsequent reflection assisted with skill development and linking theory to practice [34, 35]. It has been previously reported that the educational relevance of feedback and reflection on practice, needs to be understood to ensure protected reflection time is built into each clinical day [6, 36]. Although students in this current study were appreciative of feedback it was not commonly provided. Midwives have previously identified the need to be educated on how to provide feedback to students however they also expressed the desire for student reciprocity in the process [16, 17]. In this study midwives used positive language when giving feedback, the midwives use of positive affirmation correlates with a previous study that found that feedback is rewarding to students and encourages repeat behaviour [28].

Limitations of this study include the small number of participants from one university. Due to the small number of participants transferability of findings could also be limited, as all participants were postgraduate students of midwifery. Another limitation is that all students were postgraduate students and were already registered nurses with previous knowledge of working within a health setting. Students participating in the study are in a paid employment model which adds the complexities of balancing employment and learning demands. These demands are not experienced by undergraduate students in an unpaid model, therefore may have an impact on transferability. The study’s strengths were that participants were placed in several hospitals with a range of acuity from local district to referral hospitals, providing data contributing to further understanding of the effective strategies used by midwives to support students in the clinical leaning environment across a variety of settings.

## Conclusions

The use of AI was able to identify positive learning strategies used by midwives in the clinical practice setting. This study offers knowledge about supportive learning strategies identified by students on midwifery practice experience. Ideally, all midwives should be able to impart knowledge and skills to better support students’ professional development in the clinical learning environment. To ensure students experience exposure to consistent support from midwives, midwives need support to ensure they have the capacity and confidence to provide salient and timely learning opportunities.

### Electronic supplementary material

Below is the link to the electronic supplementary material.

Supplementary Material 1

## Data Availability

The datasets used and/or analysed during the current study are available from the corresponding author on reasonable request.

## Abbreviations

AI: Appreciative Inquiry
ANMAC: Australian Nursing and Midwifery Accreditation Council
BM: Bachelor of Midwifery
GDM: Graduate Diploma in Midwifery
