# Supplementary material for: Strategies used by midwives to enhance knowledge and skill development in midwifery students: an appreciative inquiry study
Source: BMC Nurs. 2024 Feb 23;23:137. doi: 10.1186/s12912-024-01784-5 (PMC10893605; doi:10.1186/s12912-024-01784-5)
Supplement: Supplementary file 1 — Supplementary Material 1 [file 12912_2024_1784_MOESM1_ESM.docx]

Interview guide

| ***Rapport building*** *- To begin the interview, I would like to know more about you and your experience as a Graduate Diploma in Midwifery student.* |
| --- |
| - *What most attracted you to undertake the Graduate Diploma in Midwifery?* |
| - *What are the things about being a midwifery student that you find most meaningful, valuable, challenging and exciting?* |
| ***Lead-in*** *– Take a few moments and reflect on your experience as a midwifery student in the practice setting. There will have been ups and downs, peaks and valleys, low points and high points. For now, please think back on high points.*  ***Topic questions***  Backward questions |
| - *Describe a specific time that the behavior or actions of a midwife facilitated you to experience a high point as a Graduate Diploma in Midwifery student.* |
| - *Describe what was happening, how you felt, and what made the situation possible.* |
| - *What were the skills, strengths and qualities that the midwife brought to the experience?* |
| This set of questions was repeated several times. When there were no more examples the interview progressed to the final set of questions.  Inward questions |
| - *What do you value most about the midwives that support your development in the practice setting?* |
| - *What are the strengths skills and qualities that you value the most of midwives in the practice setting?* |
| - *Describe how the support and relationships with midwives influences your work.* |
| - *Describe how the support and relationships with midwives influences your ability to develop midwifery knowledge and skills.* |
